# Failure Mechanism Research on Bending Fretting Fatigue of 6061-T6 Aluminum Alloy by Experiment and Finite Element Method

**DOI:** 10.3390/ma16114161

**Published:** 2023-06-02

**Authors:** Jun Ding, Long Yang, Wei Liu

**Affiliations:** 1 School of Mechanical Engineering, Southwest Petroleum University, Chengdu 610500, China; yl1336218000@163.com; 2 Institute of Mechanics, Chinese Academy of Sciences, Beijing 100864, China

**Keywords:** fretting fatigue, critical plane approach, 6061-T6 aluminum alloy, fatigue life

## Abstract

The fatigue failure mechanism of bending fretting for cyclic softening material 6061-T6 aluminum alloy was researched by experiment and finite element method. The influence of cyclic load on bending fretting fatigue was researched and the damage characteristics under different cycles was discussed experimentally though SEM images. In the simulation, a normal load transformation method was employed to obtain a simplified two-dimensional model used for simulating the bending fretting fatigue from a three-dimensional model. An advanced constitutive equation with the Abdel–Ohno rule and isotropic hardening evolution was transplanted into ABAQUS by UMAT subroutine to consider the ratchetting behavior and cyclic softening characteristics. The peak stain distributions under various cyclic loads were discussed. Additionally, the bending fretting fatigue lives and crack initiation locations referring to a critical volume method were estimated using the Smith–Watson–Topper critical plane approach and reasonable results were obtained.

## 1. Introduction

Under the external fatigue cyclic loads, fretting fatigue will happen because of micrometer-scale relative movements at the interface between contact bodies [1]. Fretting fatigue is widely found in a variety of engineering components, such as in riveted structures or bolted structures, bioimplant components, railway axles, aerospace, nuclear energy, and so on [2,3,4]. It results in the earlier failure and lower fatigue strength of the structure than plain fatigue does [5,6,7]. The prevalence and the severity of harm make fretting fatigue the focus of current engineering research work.

According to different types of fatigue loads, there are three types of fretting fatigue: fretting fatigue of tension–compression, torsion, and bending [8]. Most of the existing studies have focused on the fretting fatigue of tension–compression and torsion, while studies are limited on the fretting fatigue of bending [9,10,11,12]. Ebaraa et al. studied the bending fretting fatigue behaviors of Ti-6Al-4V [13]. Kubotaa et al. discussed the influencing factors of bending fretting fatigue, such as H_2_ and N_2_ [14]. The experiments of bending fretting fatigue for various materials such as 316 L austenitic stainless steel and LZ50 steel were carried out based on macro- and micromethod, and the fatigue failure characteristic was explored by observing the profile at the location of crack initiation [11,15,16,17,18]. Based on the experimental results, Jiang et al. carried out a finite element analysis of bending fretting fatigue [19], but the warping phenomenon observed in the experiment was not considered. Zhu et al. [20] proposed an equivalent normal load to obtain a simplified equivalent two-dimensional plane strain finite element model from the three-dimensional model to consider the warping phenomenon. Using the simplified two-dimensional model, the stress and strain distribution of LZ50 steel during the bending fretting fatigue process was analyzed numerically. However, LZ50 steel is a cyclic stable material. Research on the bending fretting fatigue of cyclic softening materials is still rare.

Therefore, the fatigue failure mechanism of bending fretting for cyclic softening material 6061-T6 aluminum alloy is worth researching in detail. First, the fatigue experiment of bending fretting for 6061-T6 aluminum alloy was carried out, and the damage evolution under different cycles was revealed. Then, based on the experiment, finite element simulation was conducted to analyze the peak strain distribution by using an advanced constitutive equation. In this constitutive, a nonlinear isotropic hardening equation was employed to describe cyclic softening and the Abdel–Ohno equation was introduced to describe the ratchetting. Finally, the fatigue lives and crack initiation locations were assessed using the Smith–Watson–Topper (abbreviated as SWT) critical plane approach.

## 2. Bending Fretting Experiment

### 2.1. Materials and Test Method

**Figure 1:**
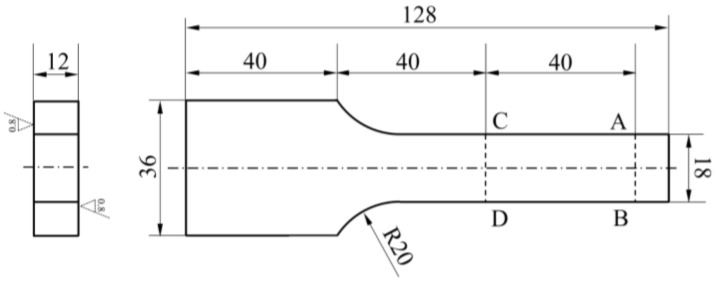
Fatigue specimen geometry of bending fretting.

The material of fatigue specimen used in the bending fretting experiment was 6061-T6 aluminum alloy. Figure 1 gives the specimen, which was created in the shape of a half dog bone. The material used for the fretting pad was 52100 bearing steel. The pad was designed to be a half cylinder and the radius was set to be 5 mm. The chemical compositions of 6061-T6 aluminum alloy: Fe, 0.7%; Ti, 0.15%; Si, 0.6%; Zn, 0.25%; Mg, 1.0%; Cu, 0.3%; Cr, 0.25%; Al, balance. The chemical compositions of 52100 bearing steel: C, 1.0%; Si, 0.25%; Mn, 0.30%; Mo, 0.05%; Cr, 1.50%; Ni, 0.20%; V, 0.15%; S, 0.03%; P, 0.027%; Fe, balance. Table 1 shows the mechanical properties of 6061-T6 aluminum alloy and 52100 bearing steel.

**Figure 2:**
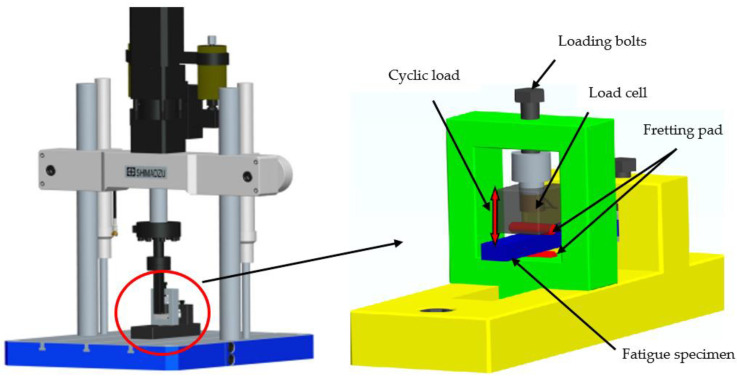
The schematic diagram of the experimental device.

The fatigue experiments of bending fretting for 6061-T6 aluminum alloy were carried out by a uniaxial fatigue machine named as EHF-UM100K2-040-OA to obtain the behavior of the bending fretting fatigue. Figure 2 shows the diagram of the fatigue experiment device. The blue plate is the fatigue specimen of 6061-T6 aluminum alloy; the two red half-cylinders are the pads of 52100 bearing steel. It should be noted that Peng et al. [16,17,18] used the point contact mode between the cylindrical specimen and cylindrical fretting pad when studying the bending fretting fatigue. In this study, the plate specimen was used to form the line contact mode, because the line contact mode has many advantages, such as good contact state, good experimental repeatability, ease of establishing finite element models and conducting mechanical analyses, and increased closeness to actual contact. The left end of the fatigue specimen in Figure 1 is fixed to the fatigue machine, and the location A-B is loaded with a cyclic bending load by the fatigue testing machine. Location C-D is the initial contact line of specimen–pad before being subjected to the cyclic bending load.

The bending fretting fatigue experiment device mainly consists of a vertical fatigue load application device, a normal load application device, and a base frame. A high-precision mechanical sensor is embedded in the normal load application device. The normal load is imposed and controlled by a load cell through bolts, and the upper pad is in contact with the fatigue specimen; the fatigue load is applied at the end of the fatigue specimen. During the experiment, the effect of bending fatigue load causes bending deformation of the fatigue specimen, resulting in microdisplacement between the fatigue specimen and the fretting pad. The plain bending fatigue with zero normal load can also be conducted by this experimental equipment. The frequency of the bending fretting experiment was selected as 20 Hz. The loading waveform was sine wave and the load ratio of cyclic load was 0.1.

### 2.2. Relation of Cyclic Load and Fatigue Lives

**Figure 3:**
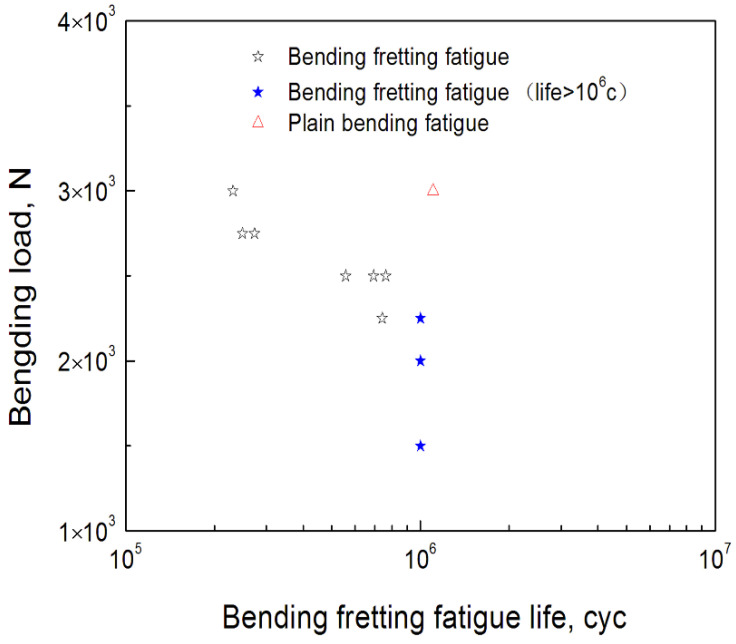
The relationship between cyclic bending loads and fatigue lives.

In order to analyze the relationship between cyclic load and fatigue lives of bending fretting, the bending fretting experiments with various cyclic bending loads and identical normal load for 6061-T6 aluminum alloy were carried out. The identical normal load was selected as 1000 N. The peak cyclic loads were prescribed as 3000 N, 2750 N, 2500 N, 2250 N, 2000 N, and 1500 N, respectively. Figure 3 shows the experimental results. It should be noted that the lives shown by blue stars with peak bending loads of 2250 N, 2000 N, and 1500 N in Figure 3 are not the actual lives, because the fatigue life limit of fretting bending in the experiments was set to be 1 × 10^6^ cycles. The fatigue lives of bending fretting for 6061-T6 aluminum alloy decreased clearly with the increase in cyclic load. This indicates that cyclic load has an obvious influence on the fatigue life of bending fretting. This conclusion is different from the research results under point contact. Under point contact mode, the bending fretting fatigue life of 7075 aluminum alloy first decreases and then increases with the increase in bending fatigue load, and finally shows a decreasing trend [16].

Moreover, the fatigue experiments of plain bending with zero normal load were conducted to compare the difference of bending fretting and plain bending. When the peak cyclic load was 3000 N, which is shown in Figure 3, the fatigue life of plain bending was clearly higher than that of bending fretting. Peng et al. [16] reached the same conclusion. Whether in point contact or line contact mode, under the same peak cyclic load, the fatigue life of plain bending is significantly longer than that of bending fretting fatigue. The high-local-contact stress of the fretting damage area caused by the cyclic load and normal load resulted in the early initiation of fatigue microcracks. Then, the fatigue cracks further expanded under the cyclic load and finally resulted in fatigue failure. This indicates that the coupling effect of cyclic load and normal load makes the fretting damage on the contact surface conducive to crack formation and further propagation, and finally results in a shortened fatigue life and the reduced fatigue strength of the structure.

### 2.3. Fracture Analysis of Bending Fretting Fatigue

**Figure 4:**
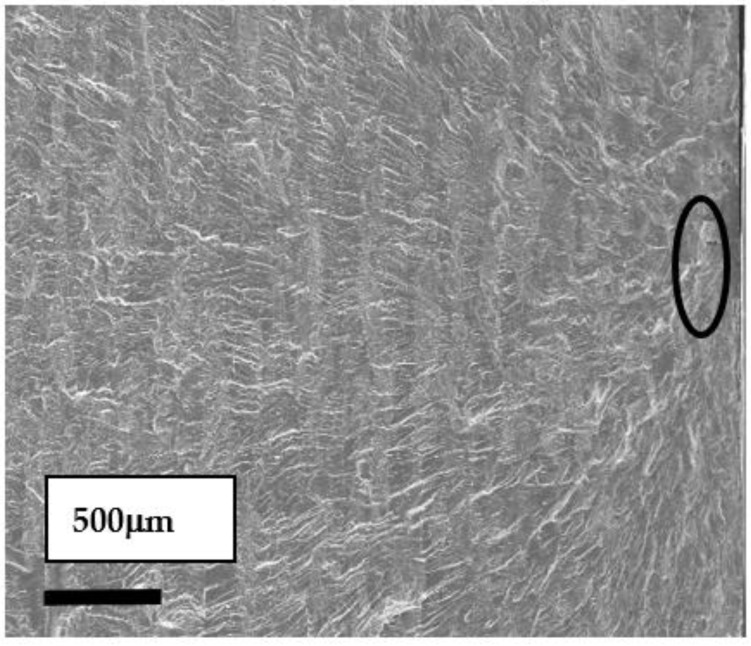
SEM images of fatigue fracture source.

The fatigue cracking of bending fretting initiated and expanded in the fretting damage area, so the fracture surface of the fretting damage region was researched. Figure 4 illustrates the fracture morphology analysis results with the normal load of 1000 N and the peak cyclic load of 3000 N. The fatigue crack source marked by the black circle in Figure 4 is seated on the upper surface of the plate specimen. The secondary fatigue crack initiation of the contact region is caused by the local high-contact stress.

**Figure 5:**
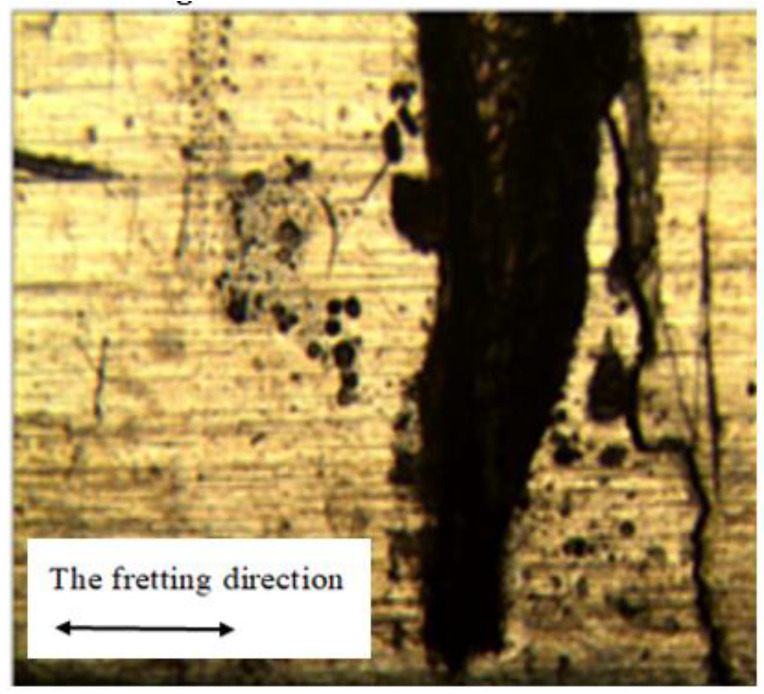
OM diagram of crack initiation position of fatigue specimen.

Figure 5 exhibits the optical microscope appearance result of the contact area for 6061-T6 aluminum alloy. The normal load was 1000 N and the peak cyclic load was 2750 N. The fatigue crack of bending fretting is located at the fretting damage area and on the right side of the original contact center.

### 2.4. Evolution of Fretting Damage under Different Cycles

**Figure 6:**
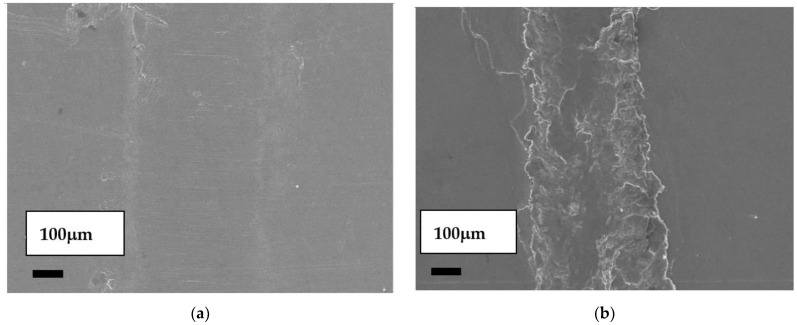
SEM images of the fretting damage area for bending fretting fatigue when (**a**) the number cycle N was 5 × 10^5^ c and (**b**) the number cycle N was 2 × 10^6^ c.

The fretting damage area under different cycles with a normal load of 1000 N and a peak cyclic load of 1500 N was discussed to reveal the damage evolution characteristics during the bending fretting fatigue process. Figure 6 shows the SEM images of the fretting damage area for 6061-T6 aluminum alloy with different cycles.

As given in Figure 6, with the cycle number of 5 × 10^5^ c, the material surface was very slightly scratched. As the cycle increased to 2 × 10^6^ c, the damaged area on the contact surface enlarged significantly, and the damage became more severe. The asperity on the surface of the fretting zone caused the material to produce corresponding plastic deformation. Under external cyclic loading, the surface material was squeezed to both sides, perpendicular to the fretting direction, and then new asperity formed. After repeated sliding, rolling, and oxidation, the plastic deformation layer on the surface of the contact area gradually hardened and became brittle. Then, part of the material detached from the surface of the specimen and formed debris. Under cyclic loading, massive peeling appeared on the debris bed and formed into a third body to cover the surface of the fatigue specimen. The debris accelerated the failure process of bending fretting fatigue.

## 3. Finite Element Simulation

### 3.1. Cyclic Constitutive Equation

#### 3.1.1. Main Equations

Under small deformation, the main governing equations are:

(1) $$\varepsilon =\varepsilon^{p}+\varepsilon^{e}$$

(2) $$\varepsilon^{e}=D^{-1}:\sigma$$

(3) $${\dot{\varepsilon }}_{}^{p}=\sqrt{\frac{3}{2}}\dot{\lambda }\frac{s-\alpha }{\left\|s-\alpha \right\|}$$

(4) $$F_{y}=\sqrt{\frac{3}{2}(s-\alpha ):(s-\alpha )}-Q_{}$$

where, ε, $\varepsilon^{p}$, and $\varepsilon^{e}$ are the total, plastic, and elastic strain tensor. D is Hooke’s elasticity tensor and σ is the stress tensor. ${\dot{\varepsilon }}_{}^{p}$ is the plastic strain rate tensor. $\dot{\lambda }$ is a scalar which is calculated by the consistency condition $\dot{\lambda }{\dot{F}}_{y}=0$. s is the deviatoric stress tensor and α is the back stress tensor. (:) is the inner product of tensors $Q_{}$ refers to the resistance of isotropic deformation.

#### 3.1.2. Nonlinear Kinematic Hardening Evolution

Plastic deformation accumulation occurs in the fatigue process of bending fretting. Therefore, the Abdel–Ohno hardening equation [21] was adopted to describe the influence of ratcheting behavior on bending fretting for 6061-T6 aluminum alloy.

The back stress tensor α of the Abdel–Ohno equation is composed as:

(5) $$\alpha =\sum_{i=1}^{M}\alpha^{\left(i\right)}$$

Each back stress $\alpha^{\left(i\right)}$ is given by the following nonlinear evolution formula:

(6) $${\dot{\alpha }}^{\left(i\right)}=\zeta^{\left(i\right)}\left[\frac{2}{3}r^{\left(i\right)}{\dot{\varepsilon }}^{p}-\mu^{\left(i\right)}\alpha^{\left(i\right)}\dot{p}-H(f^{\left(i\right)})\alpha^{\left(i\right)}\langle {\dot{\varepsilon }}^{p}:K^{\left(i\right)}-\mu^{\left(i\right)}\dot{p}\rangle \right]$$

where $r^{\left(i\right)}$ and $\zeta^{\left(i\right)}$ are material parameters and $K^{\left(i\right)}=\alpha^{\left(i\right)}/\left\|\alpha^{\left(i\right)}\right\|$. The accumulated plastic strain rate $\dot{p}$ is obtained with p˙=(32ε˙pε˙p)12 and $H(f^{\left(i\right)})$ is the Heaviside function. The ratchetting parameter $\mu^{\left(i\right)}$ for all back stress is described as identical value, i.e., $\mu^{\left(i\right)}=\mu$. The critical plane is represented as:

(7) $$f^{\left(i\right)}={\left\|\alpha^{\left(i\right)}\right\|}^{2}-{\left(r^{\left(i\right)}\right)}^{2}=0$$

#### 3.1.3. Isotropic Hardening Evolution

6061-T6 aluminum alloy is a typical cyclic softening material [22]. Therefore, the following nonlinear equation of isotropic hardening was employed to describe the influence of cyclic softening on the ratchetting:

(8) $$\dot{Q}=\gamma (Q_{sa}-Q)\dot{p}$$

where γ determines the evolution rate of Q. $Q_{sa}$ is the resistance of saturated isotropic deformation and is prescribed as a constant for simplicity.

The cyclic constitutive equation introduced above was transplanted to the finite element software ABAQUS through secondary development by UMAT. Table 2 gives the material constants of 6061-T6 aluminum alloy which can be determined in detail referring to Reference [23]. The elastic modulus of the fretting pad was assumed to be 210 GPa and Poisson’s ratio was described as 0.3. It was supposed to be a linear and elastic material in this simulation.

### 3.2. Verification of the Suggested Cyclic Constitutive Equation

In order to validate the ability of the used constitutive equation, the monotonic tensile curves of 6061-T6 aluminum alloy and the uniaxial ratcheting behavior were simulated by establishing a three-dimensional 8-node 6-faceted isoparametric element of ABAQUS.

**Figure 7:**
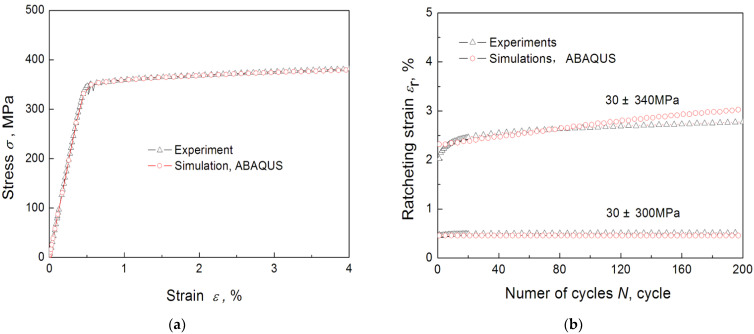
The simulated results using the new advanced cyclic constitutive equations (**a**) the tensile stress–strain curves (**b**) the uniaxial ratcheting behavior.

Figure 7 gives the simulated monotonic tensile result of 6061-T6 aluminum alloy by using the new constitutive equation introduced in the above subsection. The simulation curve is in strong agreement with the experiment curve.

The uniaxial ratchetting behaviors with different amplitudes (300 MPa and 340 Mpa, respectively) and same mean stress (30 Mpa) were carried out and Figure 7b shows the simulation results. The maximum strain is usually considered to be a limit value of fatigue failure under cyclic loading, so the maximum strain of each cycle is defined as ratchetting strain. The simulated ratchetting curves by ABAQUS are in strong agreement with the experiments.

The cyclic elastic–plastic ratcheting constitutive equation selected in this paper can describe the tension curve and ratcheting behavior of 6061-T6 aluminum alloy reasonably, and it indicates that adopting this constitutive equation is reasonable to simulate the process of bending fretting.

### 3.3. Two-Dimensional Finite Element Model

**Figure 8:**
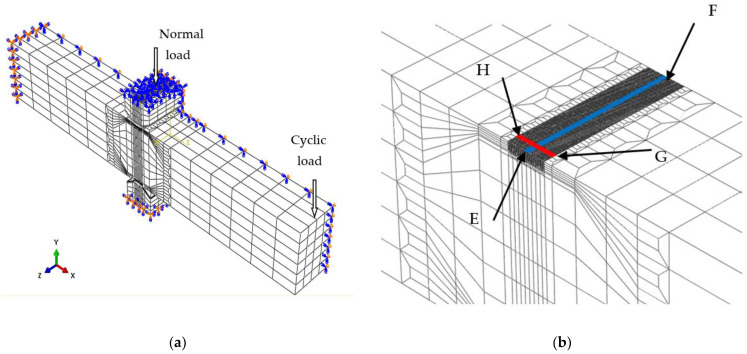
Finite element model (**a**) three-dimensional model and (**b**) the mesh.

The finite element analysis by employing a three-dimensional finite element model (referred to as 3-D model) is not suitable for revealing the characteristics of bending fretting fatigue due to the limitations of the computer. A two-dimensional plane strain finite element model (referred to as 2-D model) should be obtained through simplification. It was demonstrated that using an equivalent normal load transformation considering the warping phenomenon to obtain the 2-D model from the 3-D model is reasonable [20]. Therefore, a 3-D model of 6061-T6 aluminum alloy shown in Figure 8 was established referring to the experiment. As described in Section 2.1, the *z*-dimension of 6061-T6 aluminum alloy in the experiment should be 18 mm. Based on the symmetry of the applied load and the boundary conditions, the *z*-dimension of 6061-T6 aluminum alloy can be set to be half of the experimental size, that is, 9 mm. The symmetry plane with *z* = 0 was imposed by a symmetry constraint with *Uz* = 0 to conduct rapid and effective calculations. The specimen surface was directly controlled by the pad surface. Both were set to be a contact pair to measure the separation of the contact interface. The Coulomb model was employed to describe the interaction between two contact surfaces, and the frictional coefficient of specimen–pad was taken as a constant, i.e., 0.2. The left plane of the fatigue specimen was applied by a fixed constraint. Two pads were placed 40 mm from the left plane of the fatigue specimen. The upper pad was marked by Pad 1 and the lower pad was marked by Pad2. The plane with *z* = 0 of the pads was imposed by *Uz* = 0. The top plane for Pad 1 was applied by *Ux* = 0. The bottom plane for Pad 2 was imposed by a fixed constraint. A cyclic load was loaded on the right plane of the fatigue plate. A normal load was applied on the top plane of Pad 1. There were 61,102 nodes and 53,224 elements for the 3-D model, including 38,782 nodes and 34,728 elements for fatigue specimens, and 11,160 nodes and 9248 elements for each pad. A C3D8I element (an 8-node linear brick, incompatible modes) was used. The stress and strain distribution on the top plane for 6061-T6 aluminum alloy should be discussed in detail, because the tensile stress of the top plane is more likely to cause crack initiation and propagation. To facilitate the subsequent analysis, Path 1 and Path 2 are defined in Figure 8. The path from Point E (0, 6, 9) to the Point F (0, 6, 0) on the top plane is marked as Path 1 and it is the initial contact line of specimen–pad before applying the cyclic bending load. The path from Point H (−1.2, 6, 8.75) to Point G (1.2, 6, 8.75) is marked as Path 2.

**Figure 9:**
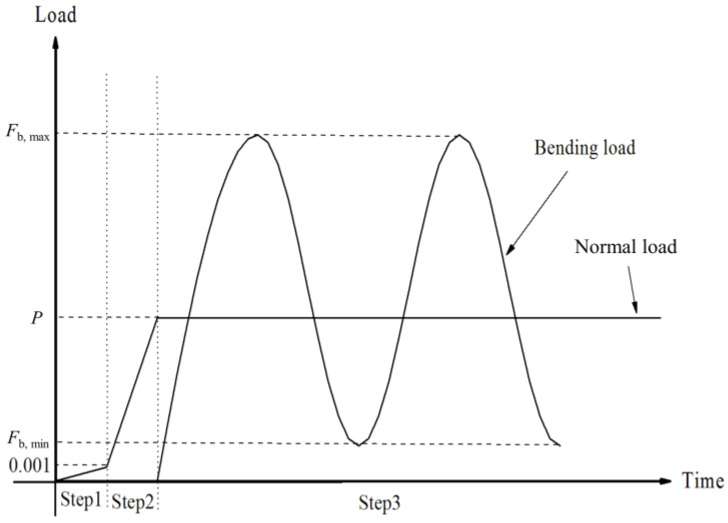
The loading process for the 3-D model.

When analyzing contact problems, the model can fail to converge if all loads are applied to the finite element model in the first analysis step. Therefore, the entire load history was divided into the following three analysis steps. First, in order to establish a more stable contact state, a smaller normal load (0.001 MPa) was applied in the y-direction to the upper surface of Pad 1. Second, the normal load was increased to the true normal load value *P* to establish a true contact. Third, the cyclic bending load *F*_b_ was applied to the right plane of the fatigue specimen to form bending fretting fatigue. The loading process described above is shown in Figure 9.

**Figure 10:**
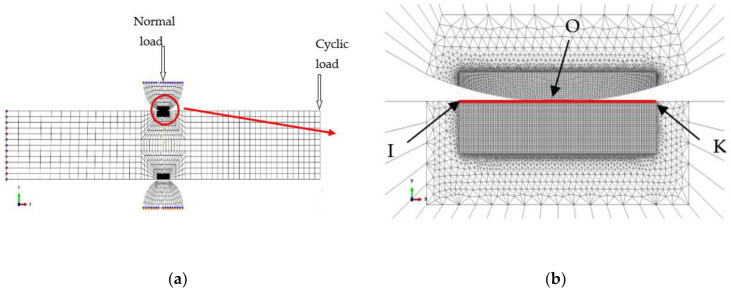
Finite element model (**a**) two-dimensional model and (**b**) the mesh.

An equivalent normal load transformation which was introduced in detail and demonstrated reasonably by Zhu et al. [20] was employed to obtain a simplified 2-D model from the calculated results of the 3-D model. Figure 10 shows the 2-D model.

This normal load transformation formula is expressed by:

(9) $$F=\mathrm{MAX}\left\{\sum_{i=1}^{n}CNORMF(z,i,t)\right\}$$

where *F* applying on the 2-D model is the equivalent normal load per unit length; *n* is the contact nodes number along Path 2; *z* is the z-coordinate along Path 1; *t* is the load substep time of each cycle; and CNORMF(z,i,t) is the normal load of the *i*-th node for the 3-D model.

Figure 10 shows Path 2 which is defined as the path from Point I (−1.2, 6) to Point K (1.2, 6) on the upper plane. Point O (0, 6) is defined as the initial contact center before applying the cyclic bending load.

The dimensions of *x* and *y* directions for the 2-D model were 80 mm and 12 mm, respectively. There were 2912 nodes and 3384 elements for the 2-D model. The size of *x* and *y* directions for the refined area was 12.5 μm and 15.625 μm, respectively. The top edge of Pad 1 was imposed by *Ux* = 0. The bottom edge of Pad 2 and the left end of the 6061-T6 aluminum alloy were imposed by fixed constraints. The normal load *F* obtained by Equation (9) should be loaded on the top edge of Pad 1. The contact conditions were the same as the description in the 3-D model. There were 25,004 nodes and 29,598 elements for the 2-D model, including 16,226 nodes and 18,570 elements for fatigue specimens, and 4389 nodes and 5514 elements for each pad. Elements with CPE3 (3-node, plane strain) and CPE4I (4-node, plane strain) were used in the 2-D model.

**Figure 11:**
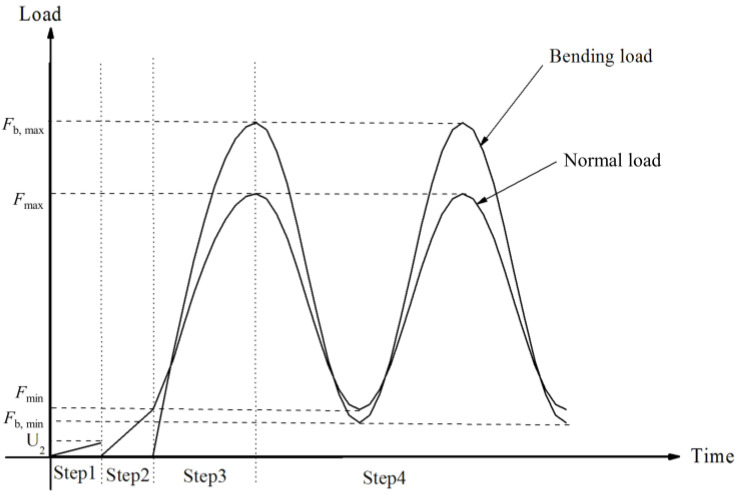
The loading process for the 2-D model.

Four loading steps were used. First, a displacement in the *y*-direction (*U*_2_ = −0.001) was loaded to the upper surface of Pad 1 to establish a relatively stable contact. Second, the normal load was increased to the minimum value *F*_min_ of the true normal load to establish a true contact. Third, the normal load was increased from the minimum value *F*_min_ to the maximum value *F*_max_, and the cyclic bending load was applied gradually to the finite element model from 0 to the peak value *F*_b, max_ of the bending load. Forth, the normal load *F* and bending load *F*_b_ of subsequent cycles were applied to the finite element model. The loading process described above is shown in Figure 11.

### 3.4. Simulation Results

The damage process of bending fretting fatigue can be understood through the experimental research, but the evolution of stress and strain in the contact area during bending fretting is not clear. Therefore, the finite element software ABAQUS should be used to simulate the bending fretting behavior by using an advanced cyclic constitutive equation to verify the ability in Section 3.2.

**Figure 12:**
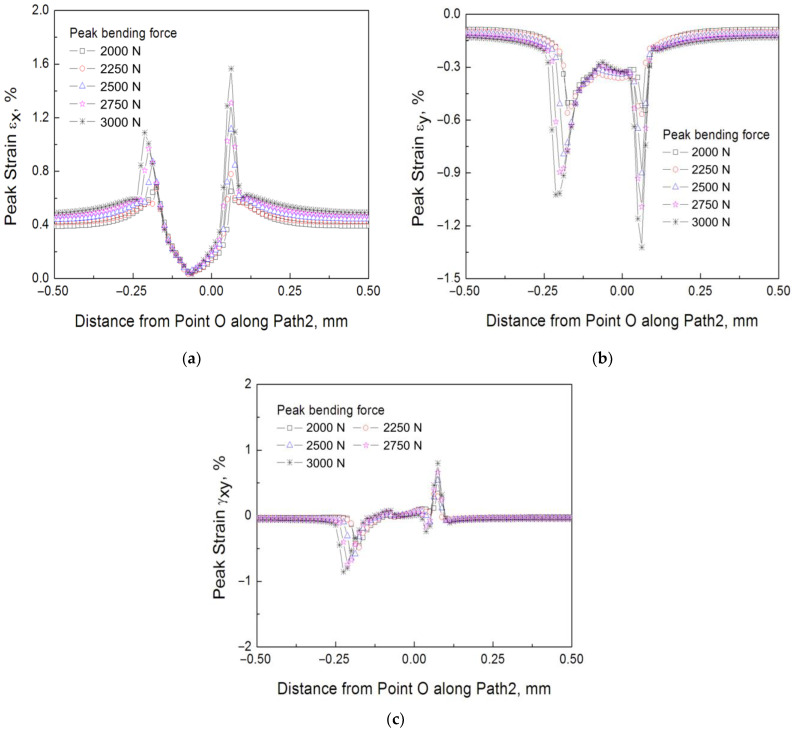
The peak strain distributions along Path 2 with various peak cyclic loads (**a**) *ε*_x_ (**b**) *ε*_y_ (**c**) *γ*_xy_.

Based on the refined 2-D finite element model introduced in Section 3.3, the bending fretting fatigue for 6061-T6 aluminum alloy with various peak cyclic loads (3000 N, 2750 N, 2500 N, 2250 N, and 2000 N, respectively) under the same normal load with 1000 N were simulated. The force ratio was prescribed as 0.1. Figure 12 illustrates the simulation results of peak strains *ε*_x_, *ε*_y_, and *γ*_xy_ along Path 2 in the 100th cycle. From these figures, the following conclusions can be drawn. First, two extreme points exist on two sides of Point O for all the distributions of *ε*_x_, *ε*_y_, and *γ*_xy_. Point O is defined in Section 3.3. It demonstrates that there is a partial slip of specimen–pad in the fretting process which is in strong agreement with the experiments. Second, the lower extreme values of strains *ε*_x_, *ε*_y_, and *γ*_xy_ on the left side of Point O suggest that crack initiation occurs on the right of the contact center, which is consistent with the experiment. Third, as the peak value of cyclic loading increases, the strains *ε*_x_, *ε*_y_, and *γ*_xy_ on both sides of Point O grow which causes early crack initiation and a short bending fretting fatigue life. Moreover, there is a slight shift of the extreme points located on both sides of Point O which is similar to the cyclic stable material such as LZ50 steel [20].

## 4. Prediction of Fatigue Life

In recent decades, the SWT critical plane approach has been used by more and more researchers to estimate the crack initiation of fretting fatigue. This method indicates that the fatigue crack initiation occurs in the plane where the maximum *SWT* parameter takes place. In this work, the *SWT* critical plane approach was introduced to estimate the bending fretting fatigue lives and the crack initiation locations for cyclic softening material 6061-T6 aluminum alloy.

The damage parameter of the *SWT* approach is defined by the following equation:

(10) $$SWT=\sigma_{\max}\varepsilon_{a}=\frac{\sigma_{f}^{\prime 2}}{E}{\left(2N_{f}\right)}^{2b}+\sigma_{f}^{\prime }\varepsilon_{f}^{\prime }{\left(2N_{f}\right)}^{b+c}$$

where $\sigma_{\max}$ is the maximum normal stress, $\varepsilon_{a}$ is the strain range, and E is the Elastic modulus. b, c, $\sigma_{f}^{\prime }$, and $\varepsilon_{f}^{\prime }$ are the fatigue strength exponent, fatigue ductility exponent, fatigue strength coefficient, and fatigue ductility coefficient, respectively. The value of these four material constants b, c, $\sigma_{f}^{\prime }$, and $\varepsilon_{f}^{\prime }$ for 6061-T6 aluminum alloy should be determined from the plain fatigue by referring to Reference [24]. They are −0.085, −1.063, 573.8 MPa, and 2.912.

**Figure 13:**
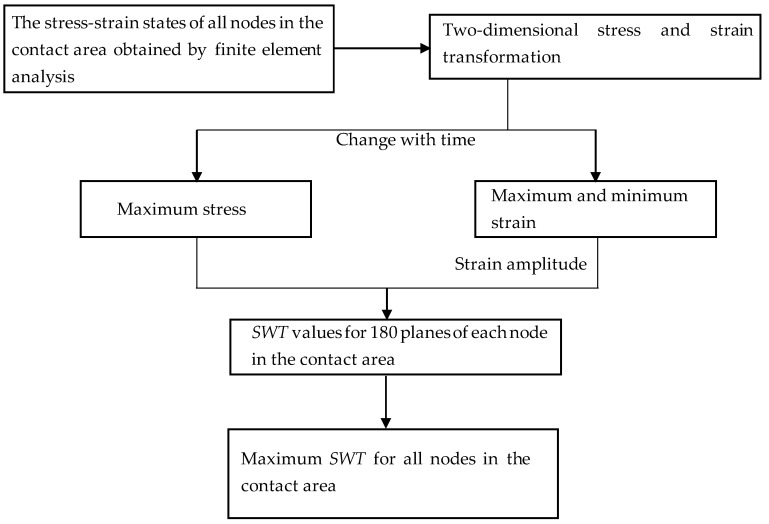
Procedure to find the maximum *SWT* value using the critical plane method.

**Figure 14:**
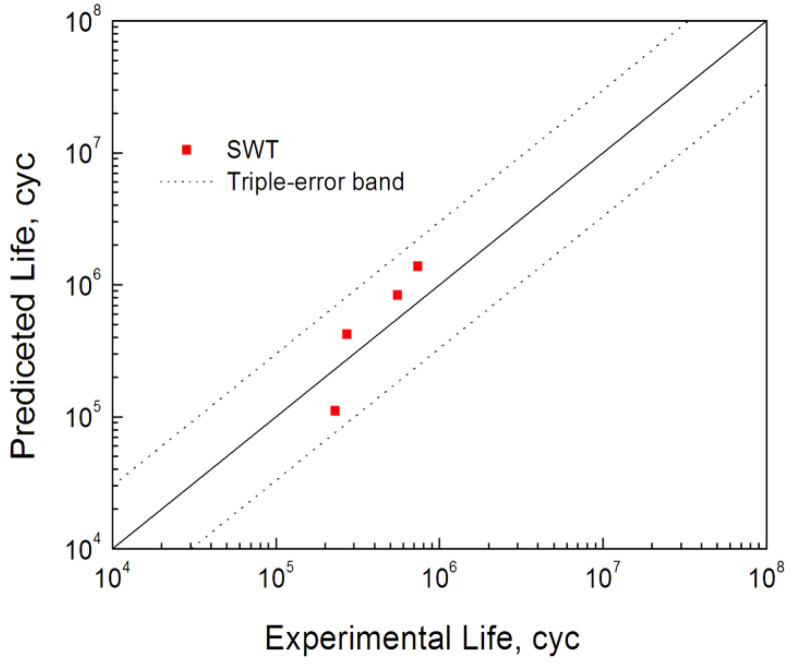
Predicted lives of bending fretting fatigue with the same normal load and various cyclic loads.

Considering the influence of ratchetting behavior and to save calculation time, the results of the 100th cycle were selected for the fatigue life prediction of bending fretting. Based on the calculated stress–strain values for all nodes along Path 2 under various peak cyclic loads and the identical normal force, the maximum *SWT* values along Path 2 can be calculated. Figure 13 shows the process of calculating the maximum value of *SWT*. Then, according to the location with the largest *SWT* value, the predicted crack initiation positions are about 0.1 mm to the right of the original contact center for all loading cases which are consistent with the experimental results. The obtained maximum *SWT* parameter is substituted into Equation (10), and the fatigue lives of bending fretting were calculated referring to a volume average method [25]. The critical volume *V*_c_ is about one grain volume, taken as 1.25 *l*_c_ × *l*_c_ × unit thickness in *z* direction. The grain length *l*_c_ of 6061-T6 aluminum alloy was about 60 μm. Figure 14 exhibits the estimated fatigue lives of bending fretting. All the results are located within the triple error band. It demonstrates that the estimated fatigue lives of 6061-T6 aluminum alloy are consistent with the experiments.

## 5. Conclusions

(1) The fatigue life of plain bending with zero normal load is clearly higher than that of bending fretting under the same cyclic bending load.
(2) The bending fretting fatigue lifespan of 6061-T6 aluminum alloy decreases significantly with the increase in peak bending force under the same normal load. So, the bending load is an important factor that cannot be ignored in analyzing bending fretting fatigue failure.
(3) Based on the calculated finite element results, the bending fretting fatigue lives and crack initiation positions can be assessed reasonably using the *SWT* critical plane approach referring to a critical averaging dimension method.

## Figures and Tables

**Table 1:** Mechanical properties of the two materials used.

Materials	Yield Stress *σ*_0.2_ (MPa)	Strength Limit *σ*_b_ (MPa)	Hardness HV	Elastic Modulus *E* (GPa)
6061-T6 aluminum alloy	295	324	95	76
52100 bearing steel	1700	2000	890	210

**Table 2:** Material constant for 6061-T6 aluminum alloy used in the simulation.

*M* = 8, *μ* = 0.01, *γ* = 4.0; *E* = 76 GPa, *Q*_0_ = 315.8 MPa, *Q*_sa_ = 310.8 MPa, *v* = 0.33, *ζ*^(1)^ = 10,000, *ζ*^(2)^ = 2083, *ζ*^(3)^ = 649, *ζ*^(4)^ = 359.7, *ζ*^(5)^ = 207, *ζ*^(6)^ = 101, *ζ*^(7)^ = 50.8, *ζ*^(8)^ = 25; *r*^(1)^ = 2.51, *r*^(2)^ = 25.8, *r*^(3)^ = 4.39, *r*^(4)^ = 2.3, *r*^(5)^ = 1.68, *r*^(6)^ = 3.94, *r*^(7)^ = 3.92, *r*^(8)^ = 22.66 MPa

## Data Availability

Not applicable.

## Nomenclature

σ_0.2_	yield stress
HV	hardness
ε	total strain tensor
$\varepsilon_{}^{e}$	elastic strain tensor
σ	stress tensor
${\dot{\varepsilon }}_{}^{p}$	plastic strain rate tensor
α	back stress tensor
Q	isotropic deformation resistance
$\alpha^{\left(i\right)}$	back stress component
μ,$\mu^{\left(i\right)}$	ratchetting parameter
$\dot{p}$	accumulated inelastic strain
$f^{\left(i\right)}$	critical surface
*v*	Poisson’s ratio
F	equivalent normal force per unit length applied in the 2-D finite element model
$N_{f}$	fatigue life
$\varepsilon_{a}$	strain range
c	fatigue ductility exponent
$\varepsilon_{f}^{\prime }$	fatigue ductility coefficient
*l*_c_	the length of cubic grain
σ_b_	strength limit
E	elastic modulus
$\varepsilon^{p}$	plastic strain tensor
D	Hooke’s elasticity tensor
(:)	inner product of tensors
s	deviatoric stress tensor
$F_{y}$	yield function
$r^{\left(i\right)}$,$\zeta^{\left(i\right)}$	material constants for kinematic hardening
$K^{\left(i\right)}$	direction tensor of i-th back stress
$H(f^{\left(i\right)})$	Heaviside function
$Q_{sa}$	resistance of saturated isotropic deformation
γ	material parameter that controls evolution rate
*ε*_x_, *ε*_y_, *γ*_xy_	peak strain components
$\sigma_{\max}$	maximum normal stress
b	fatigue strength exponent
$\sigma_{f}^{\prime }$	fatigue strength coefficient
*V*_c_	critical volume
